# First Insights into the Genome of the *N*-Methylhydantoin-Degrading *Clostridium* sp. Strain FS41 (DSM 6877)

**DOI:** 10.1128/genomeA.00394-15

**Published:** 2015-04-30

**Authors:** Anja Poehlein, Adrian Bandera, Deborah Horne, Joachim Maier, David Pawlowicz, Verena Siebert, Rolf Daniel

**Affiliations:** a Genomic and Applied Microbiology & Göttingen Genomics Laboratory, Institute of Microbiology and Genetics, Georg-August University, Göttingen, Germany; b Members of the Applied Bioinformatics in Microbiology Course of the Microbiology and Biochemistry MSc/PhD program, Georg-August University, Göttingen, Germany

## Abstract

*Clostridium* sp. strain FS41 (DSM 6877) is a strictly anaerobic and Gram-positive spindle-shaped rod. This spore-forming bacterium is able to degrade *N*-methylhydantoin, with *N*-carbamoylsarcosine and sarcosine as intermediates. The genome consists of one replicon (6.28 Mb) and harbors 5,735 predicted protein-coding genes.

## GENOME ANNOUNCEMENT

The *N*-methylhydantoin-degrading *Clostridium* sp. strain FS41 is a strictly anaerobic and Gram-positive spindle-shaped rod (1). This spore-forming organism has been isolated from anaerobic sludge samples derived from the sewage plant of Brebach (Brebach-Fechingen, Germany). *Clostridium* sp. FS41 (DSM 6877) was obtained from the German Collection of Microorganisms and Cell Cultures (DSMZ) (Braunschweig, Germany). Strain FS41 was cultivated under anaerobic conditions, with *N*-methylhydantoin as a substrate (1). Chromosomal DNA was isolated using the MasterPure complete DNA purification kit (Epicentre, Madison, WI, USA). Illumina shotgun sequencing libraries were generated from the extracted DNA, and sequencing was performed with a Genome Analyzer IIx, as recommended by the manufacturer (Illumina, San Diego, CA, USA). Sequencing resulted in 9,320,068 reads that were trimmed using Trimmomatic 0.32 (2). The assembly was performed with the SPAdes genome assembler software 3.5.0 (3). The assembly resulted in 97 contigs (>500 bp) and an average coverage of 154-fold. The draft genome of *Clostridium* sp. FS41 consists of 6.28 Mb, with an overall G+C content of 49.36%. The genome probably consists of a single chromosome, as evidence for the presence of plasmids, such as genes that are characteristic of plasmid replication systems, was not found. Automatic gene prediction was performed by using the software tool Prodigal (4). Genes coding for rRNA and tRNA were identified using RNAmmer (5) and tRNAscan (6), respectively. The Integrated Microbial Genomes-Expert Review (IMG-ER) system (7) was used for automatic annotation, which was subsequently manually curated by using the Swiss-Prot, TrEMBL, and InterPro databases (8). The draft genome harbored at least 3 rRNA genes, 44 tRNA genes, 4,321 protein-coding genes with predicted functions, and 1,414 genes coding for hypothetical proteins. Two intact prophage regions were encountered by using the phage search tool PHAST (9). During *N*-methylhydantoin degradation, *N*-carbamoylsarcosine and sarcosine are formed as intermediates by *Clostridium* sp. FS41. The key enzymes for this pathway are *N*-methylhydantoin amidohydrolase and *N*-carbamoylsarcosine amidohydrolase. In contrast to *N*-methylhydantoin amidohydrolase from aerobic bacteria, the enzyme of *Clostridium* sp. FS41 does not require ATP for enzymatic activity (1). A putative gene coding for *N*-carbamoylsarcosine amidohydrolase, which catalyzes the conversion of *N*-carbamoylsarcosine to sarcosine, is present in the genome. The known *N*-methylhydantoin amidohydrolases usually comprise two subunits (10), but in the genome sequence of *Clostridium* sp. FS41, only the gene encoding subunit B was identified. The further reduction of sarcosine to acetylphosphate is performed by sarcosine reductase, a three-subunit enzyme (11). Corresponding genes were present in *Clostridium* sp. FS41 and divided into two clusters. One included the genes coding for the sarcosine-specific subunit B and a hypothetical protein, which is usually associated with these genes. The second cluster harbors the genes coding for the substrate-unspecific subunits A and C. In *Clostridium litorale* DSM 5388 (12), *Sporomusa ovata* DSM 2662 (13), and *Eubacterium acidaminophilum* DSM 3953 (14), subunit C is encoded by two genes, whereas in *Clostridium* sp. FS41, both genes are fused.

### Nucleotide sequence accession numbers.

This whole-genome shotgun project has been deposited at DDBJ/EMBL/GenBank under the accession no. JYHN00000000. The version described here is version JYHN01000000.
